# Green Channel Guiding Denoising on Bayer Image

**DOI:** 10.1155/2014/979081

**Published:** 2014-03-11

**Authors:** Xin Tan, Shiming Lai, Yu Liu, Maojun Zhang

**Affiliations:** College of Information System and Management, National University of Defense Technology, Changsha, Hunan 410073, China

## Abstract

Denoising is an indispensable function for digital cameras. In respect that noise is diffused during the demosaicking, the denoising ought to work directly on bayer data. The difficulty of denoising on bayer image is the interlaced mosaic pattern of red, green, and blue. Guided filter is a novel time efficient explicit filter kernel which can incorporate additional information from the guidance image, but it is still not applied for bayer image. In this work, we observe that the green channel of bayer mode is higher in both sampling rate and Signal-to-Noise Ratio (SNR) than the red and blue ones. Therefore the green channel can be used to guide denoising. This kind of guidance integrates the different color channels together. Experiments on both actual and simulated bayer images indicate that green channel acts well as the guidance signal, and the proposed method is competitive with other popular filter kernel denoising methods.

## 1. Introduction

Image denoising is one of the hottest spots in image processing. It can not only enhance image quality but also increase compression efficiency and improve the robustness of the subsequent intelligent analysis algorithms, such as objection detection and pattern classification. Many high-performance algorithms are proposed. However, they often suffer heavy computational burden. For example, Non-Local Means (NLM) [1], BM3D [2, 3], and K-SVD [4] are based on local similar patches search; some others are based on statistical learning or dictionary training [4–6] which requires iteration optimization; some also relate to domain transformation [7, 8]. For the digital cameras, especially video cameras, the frame rate is required for 24 fps at least. Additionally, there are many image processing steps in the camera including denoising, demosaicking, automatic white balance, automatic exposure, gamma correction, color correction, brightness and contrast adjustment, and edge enhancement. All these steps need to be computed for less than 42 ms (1/24 fps). For some high-end cameras, this demand is much stricter, for example, at least 60 fps for motion-capture cameras. Thus those block matching, domain transformation, or model training based algorithms are not suitable for real time image processing in digital cameras.

Therefore, the digital camera denoising often uses the explicit filter kernel, such as mean filter, median filter, Gaussian filter, and more effective edge-preserving filter: bilateral filter [9]. In addition, based on bilateral filter, He et al. [10] proposed a new type of explicit image filter with the name guided filter. It incorporates additional information from a given guidance image. The filtered image has the same edge information as the guidance image. Guided filter has the edge-preserving property like bilateral filter but does not suffer from the gradient reversal artifacts. Moreover, it has higher time efficiency than bilateral filter [10].

**Figure 1:**
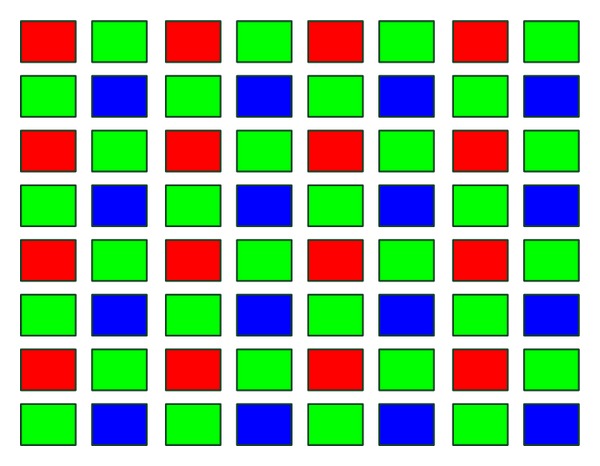
Bayer pattern color matrix with red-green-red-green phase in the first row.

Now consider the image sensor in digital cameras. Most cameras use single sensor with a color matrix. And most sensors adopt the RGB three-color bayer pattern which is shown in Figure 1.

The image which is obtained by bayer pattern sensor is called bayer image. One pixel can only record a single color, so the full three-color representation should be reconstructed by estimating the missing components from the adjacent pixels. This process is called demosaicking. Researches have focused on this interpolation problem for a long time [11–13].

At present, almost all denoising algorithms [1–10] are aiming at monochromatic images or color images. Because of the red, green, and blue interlaced pattern, these denoising methods cannot work directly on bayer image. There are two options to handle this problem. One is to remove noise from the demosaicked color image. However, this is not a good choice due to the reason that noise is spread across the channels during the demosaicking and its characteristics are more complicated. Removing noise after demosaicking causes the denoising to be more difficult [5, 14]. The other option is to denoise before demosaicking. Many algorithms that adopt this option are presented [5, 14–17]. The aim of this paper is also to design a denoising method for bayer image. In view of the fact that guided filter is an excellent filter kernel with high time efficiency, we concentrate on how to use this filter in our work.

During our research, we observe that green channel of bayer image not only has higher sampling rate than red and blue channels, but also its photosensitivity, that is, ISO, at most cases is higher. Another research [18] shows that image Signal-to-Noise Ratio (SNR) is increased with ISO at the same exposure time. Thus for guided filter, green channel can act well as a guidance signal. Thus, we propose a high SNR channel guided denoising method with guided filter.

The rest of the paper is structured as follows.  Section 2 briefly introduces the guided filter.  Section 3 presents the characteristics of bayer image's green channel and describes the algorithm flow. In Section 4, experiments on real bayer image and Kodak test sets demonstrate the efficiency of the proposed method. Finally, conclusions are drawn in Section 5.

## 2. Guided Filter Overview

In some cases, we need to merge extra information into the original image. For example, in colorization the output chromatic channels require having the same edge as the luminance channel. He et al. [10] propose an explicit filter kernel to solve this problem.

Define the guidance image *I*, the input image *p*, and the output image *q*. The assumption of guided filter is the local linear property between the guidance image and the output image. Namely, in a local window *ω*_*k*_ centered at the pixel *k*, the output *q* at a pixel *i* is

(1) qi=akIi+bk, ∀i∈ωk,

where (*a*_*k*_, *b*_*k*_) are the constant linear coefficients in *ω*_*k*_. The local window *ω*_*k*_ is a square area with the radius *r*. From (1) we know ∇*q*_*i*_ = *a*_*k*_∇*I*_*i*_. Namely, edge information of the output image is linear with that of the guidance image. In order to determine the linear coefficients (*a*_*k*_, *b*_*k*_), it should minimize the following cost function:

(2) $$\begin{array}{c} E\left(a_{k},b_{k}\right)=\underset{i\in \omega_{k}}{\sum }\left({\left(a_{k}I_{i}+b_{k}-p_{i}\right)}^{2}+\varepsilon a_{k}^{2}\right). \end{array}$$

Here *ε* is a regularization parameter preventing *a*_*k*_ from being too large. The solution of (2) is

(3) $$\begin{array}{c} a_{k}=\frac{\left(1/\left|\omega \right|\right)\sum_{i\in \omega_{k}}I_{i}p_{i}-\mu_{k}{\bar{p}}_{k}}{\sigma_{k}^{2}+\varepsilon }=\frac{\mathrm{cov}\left(I_{k},p_{k}\right)}{\sigma_{k}^{2}+\varepsilon }\\ b_{k}={\bar{p}}_{k}-a_{k}\mu_{k}. \end{array}$$

Here *μ*_*k*_ and *σ*_*k*_^2^ are the mean and variance of *I*_*k*_ in *ω*_*k*_. |*ω*| is the number of pixels in *ω*_*k*_. ${\bar{p}}_{k}$ is the mean of *p*_*k*_ in *ω*_*k*_. cov⁡(*I*_*k*_, *p*_*k*_) is the covariance between *I*_*k*_ and *p*_*k*_.

According to (1) and (3), the output can be computed as

(4) $$\begin{array}{c} q_{i}=\frac{\mathrm{cov}\left(I_{k},p_{k}\right)}{\sigma_{k}^{2}+\varepsilon }\left(I{}_{i}-\mu_{k}\right)+\bar{p}{}_{k}. \end{array}$$

In (4), there is only one parameter *ε*. Increasing *ε*, the gradient of output image is reduced. When *ε* → *∞*, $q_{i}\to {\bar{p}}_{k}$. The guided filter deteriorates to mean filter. Thus *ε* is the criterion of smoothness or sharpness. In another case, when *I* = *p*, that is, taking the input image itself as guidance, and without smoothness (*ε* = 0), then cov⁡(*I*_*k*_, *p*_*k*_) = *σ*_*k*_^2^, $\mu_{k}={\bar{p}}_{k}$, so *q*_*i*_ = *I*_*i*_. It means that the guided filter deteriorates to show itself.

Due to the fact that pixel *i* is involved in many windows and coefficients in each window are different, the output of *q*_*i*_ is different. He et al. [10] take a simple strategy that is averaging all the possible values as

(5) $$\begin{array}{c} q_{i}=\frac{1}{\left|\omega \right|}\underset{k:i\in \omega_{k}}{\sum }\left(a_{k}I_{i}+b_{k}\right)={\bar{a}}_{i}I_{i}+{\bar{b}}_{i}, \end{array}$$

where ${\bar{a}}_{i}=\left(1/\left|\omega \right|\right)\sum_{k\in \omega_{k}}a_{k}$, ${\bar{b}}_{i}=\left(1/\left|\omega \right|\right)\sum_{k\in \omega_{k}}b_{k}$.

However, this manipulation makes ∇*q* no longer scaling of ∇*I*. Since $\left({\bar{a}}_{i},{\bar{b}}_{i}\right)$ is the average of (*a*_*k*_, *b*_*k*_), it can still have $\nabla q_{i}\approx {\bar{a}}_{i}\nabla I_{i}$. Therefore guided filter is good at edge-preserving and has no gradient reversal problem as bilateral filter.

For time efficiency, He et al. [10] analyze that the time complexity is independent of the window radius *r* thanks to the utilization of the box filter, which is described in their paper. As for bilateral filter, its computational complexity increases when the kernel becomes larger. So the speed of guided filter is faster than that of bilateral filter.

In fact, guided filter is the advance of joint bilateral filter (JBF) [21]. It employs the extra guidance information to improve the filter performance too. But it suffers gradient reversal artifacts.

## 3. Green Channel Guiding Denoising

When using guided filter as a denoising method on bayer image, the key is to obtain the appropriate guiding signals. One simple scheme is treating each color channel as a subimage of gray scale and then denoising on them separately. This solution, however, does not exploit the spectral correlation among channels. The scheme that can make full use of the interchannel correlation is ideal. Moreover, the high quality guiding signal needs to have less noise, namely, higher SNR than the guided image. It also should be simple, owing to the real time and low resource consumption requirements.

According to the bayer color matrix in Figure 1, the information of the green channel is half of the entire sensor and two times of the red or blue channel. During demosaicking process, the rest three-fourths of the full image in the red or blue channel is estimated, while for the green channel only half of the full image is estimated. So the estimation error of the green channel is less than that of the other two. Furthermore, the green color can perceive brightness well, and human eye is more sensitive to brightness than chromaticity, so the green channel is more sensitive for human compared to red and blue. Owing to this reason many demosaicking methods recover the full-resolution green channel at first and then reconstruct the red and blue channel from the green one [12, 22, 23].

On the other hand, Hasinoff et al. in their research [18] found that at a given brightness and exposure time images with higher ISO have higher SNR. In their noise model, they defined the scene brightness as *ϕ*. Thus for dark pixels, additive noise is dominant, so SNR increases with *ϕ*, and, for bright pixels, photon noise is dominant, so SNR increases with $\sqrt{\phi }$. Namely, the brighter the scene is, the higher the SNR is. In [18], Hasinoff et al. improve ISO to increase the brightness, so as to raise SNR.

According to Lambertian reflection model [24], the pixel value *f*(*x*) on spatial point *x* relies on the integration of the incident ray spectral distribution *e*(*λ*), the surface reflectivity function *s*(*x*, *λ*) at point *x* of wavelength *λ*, and the sensor sensitivity function *c*(*λ*) as follows:

(6) $$\begin{array}{c} f\left(x\right)=\overset{}{\underset{\omega }{\int }}e\left(\lambda \right)c\left(\lambda \right)s\left(x,\lambda \right)d\lambda . \end{array}$$

**Figure 2:**
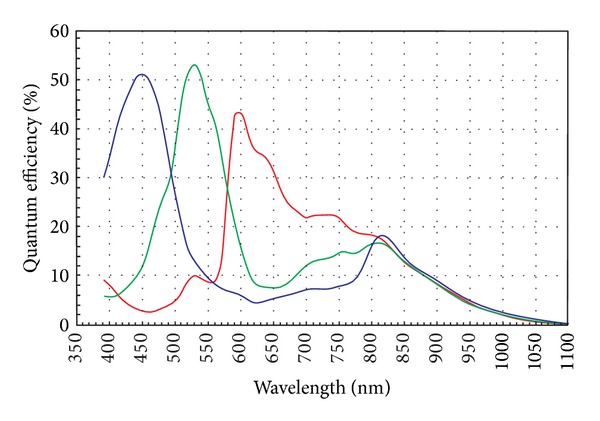
Spectral characteristics of Aptina MT9P031 CMOS sensor [19].

Here *x* is the spatial coordinates, *λ* is the spectral wavelength of the light, and *ω* is the entire visible spectrum range (wavelength from 380 nm to 780 nm). Obviously, the red, green, and blue photosensitive diodes have the different sensitive wavelength range as shown in Figure 2. This means that sensor sensitivity *c*(*λ*) of each kind of diodes is unique.

Thus the different kind of color pixels can measure the different brightness values even though the spectral distribution *e*(*λ*) and the reflectivity *s*(*x*, *λ*) are identical at the same local area. It indicates that the ISO of different color channel is different at the same circumstance for the different photo diodes sensitive characteristics.

**Figure 3:**
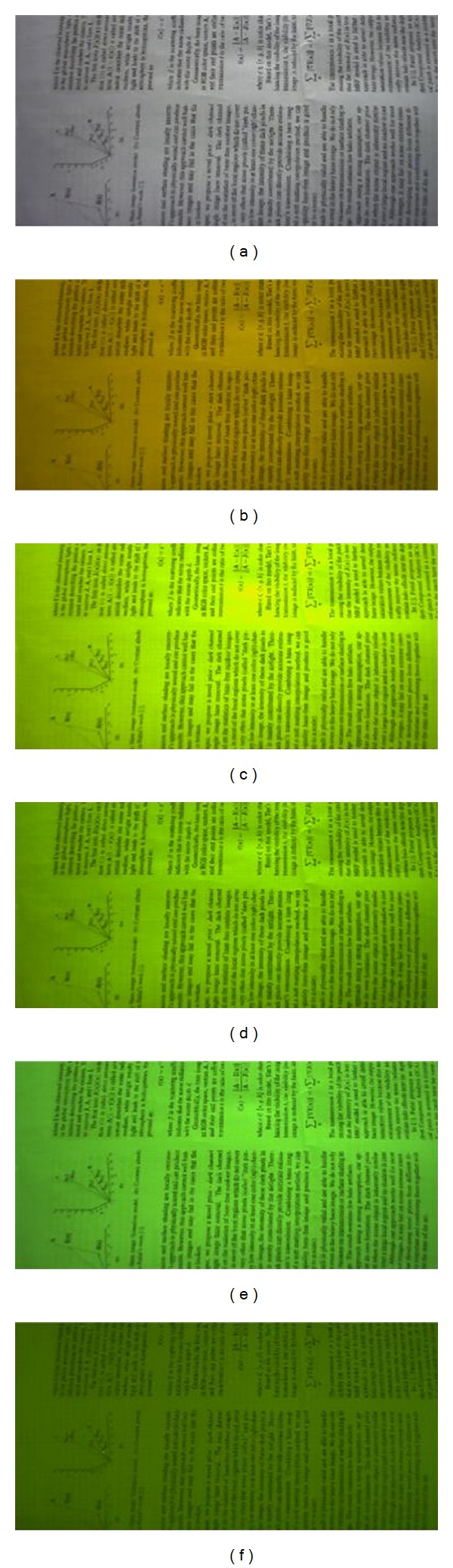
Black-and-white images under different color lights; (a) image with automatic white balance (AWB) to show the truth image, (b)–(e) images without AWB in color temperature: F 2700 K, L84 4000 K, D50T 5000 K, and D65T 6500 K, and (f) image without AWB in natural light.

Figure 3 shows the captured black-and-white images in different color lights using Aptina MT9P031 sensor. The mean brightness of Figure 3 is computed in Table 1.

**Figure 4:**
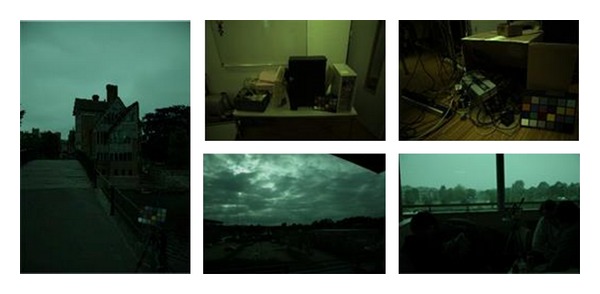
Original image (without AWB) captured by Canon SLR camera.

Figure 3 and Table 1 show that the green channel of bayer image is brighter than the others, except for the lowest (F) color temperature. It means that, in most illumination conditions, green dominates the original bayer image. Therefore, green channel possesses higher ISO than the red or blue channels at the same exposure time. The original images captured by Canon single lens reflex (SLR) camera at indoor and outdoor [25], as shown in Figure 4, can also illustrate high ISO of green channel. Thus according to Hasinoff et al.'s conclusion that at the same exposure time high ISO images have higher SNR [18], green channel has higher SNR characteristic. Based on its high sampling rate and high SNR features, the green channel can be used as guidance signal.

**Figure 5:**
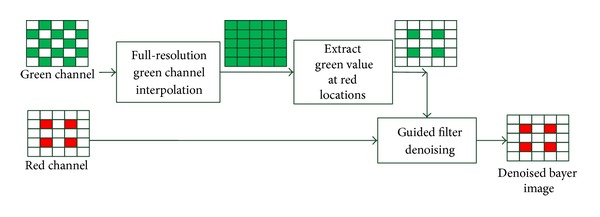
Flow chart of red channel denoising with green channel's guidance.

In order to employ green channel as the guidance image, the green value at red and blue locations should be interpolated at first. Then the guided filter is operated. The flow chart of red channel denoising is described in Figure 5. The blue channel follows the same approach. The green channel takes itself as guidance image.

**Figure 6:**
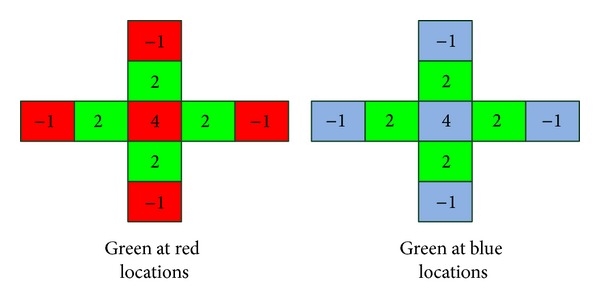
Filter coefficients for green interpolation.

As for green channel interpolation, many methods are proposed. We select a simple strategy as presented in [13] which is also chosen by Matlab image toolbox for demosaicking. The filter coefficients kernel of [13] is shown in Figure 6.

So the interpolated green value is

(7) Gr(i,j)=(2(g(i−1,j)+g(i+1,j)+g(i,j+1)+g(i,j−1))+4r(i,j)−r(i−2,j)−r(i+2,j)−r(i,j+2)−r(i,j−2))/8Gb(i,j)=(2(g(i−1,j)+g(i+1,j)+g(i,j+1)+g(i,j−1))+4b(i,j)−b(i−2,j)−b(i+2,j)−b(i,j+2)−b(i,j−2))/8,

where (*i*, *j*) is the spatial coordinates, *G*_*r*_ is the green value at red locations, and *G*_*b*_ is the green value at blue locations.

According to (7) the green interpolation correlates the red and blue channels with the green channel. Furthermore, green color is the guidance for all color channels, so denoising is unified as a whole by green channel.

## 4. Experiments and Analysis

In this section, the performance of the proposed method is tested on the actual bayer image and Kodak losses true color image suite [20]. Two aspects are examined. One is the guiding signal selection; the other is the comparison with other denoising methods.

### 4.1. Guiding Signals Comparison

In order to show the advantage of green channel guidance, other three guiding signals are compared: red channel, blue channel, and each of the channels guiding themselves. The test image is the actual bayer image of Aptina MT9P031 sensor. The reason why the bayer image recovered from the processed RGB picture is not selected is that the nonlinear image processing influences the original features of bayer image. For example, AWB destroys the high SNR characteristics of green channel, and demosaicking diffuses the dotted noise to patches, and so forth.

**Figure 7:**
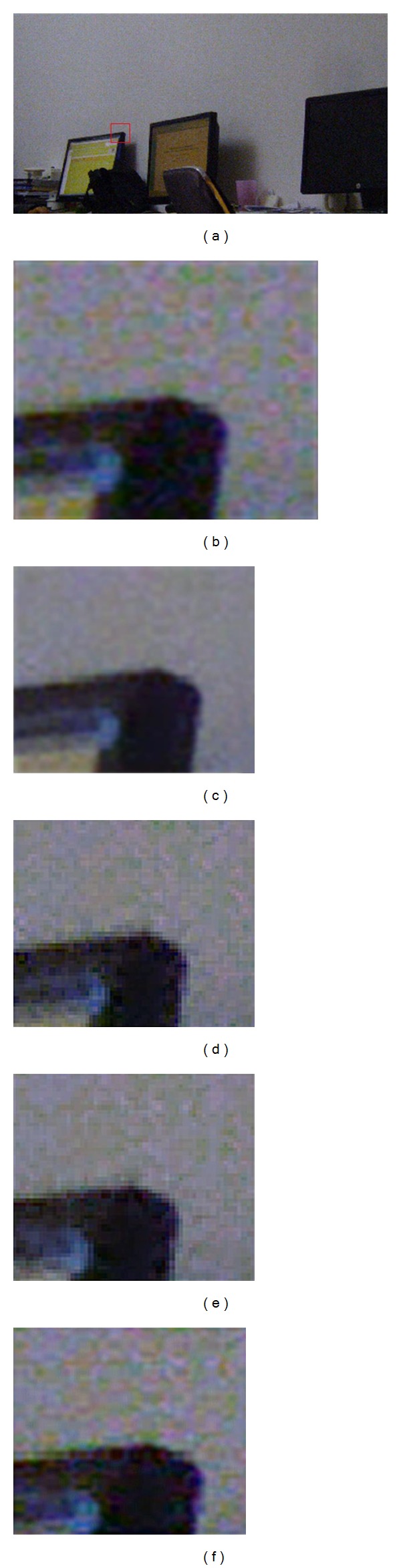
The denoising results of different guiding signals on the actual bayer image: (a) full original image, (b) an enlarged patch of (a), (c) denoised by green channel guidance, (d) denoised by red channel guidance, (e) denoised by blue channel guidance, and (f) denoised by each of the channels guiding themselves.

In our experiment, the local window radius *r* = 2, and the regularization parameter *ε* chooses the best one for each guiding signal. The interpolation for each color channel takes the method in [13].  Figure 7 is the comparison result. It shows that green channel guidance obtains the best result compared to other guiding signals. Therefore the excellent properties (high sampling rate, high ISO, and high SNR) of green channel really play an important role in guided filter denoising.

Another phenomenon is no matter the guiding image is red, blue, or green, the denoised results are all better than each of the channels guiding themselves (Figure 7(f)). The reason is that the correlation among channels is not considered when treating each color channel as an independent subimage. Guided by one color channel, the others may have the same gradients. In fact, all color channels certainly have the same gradients. Each channel guiding itself can result in different gradients in noise area.

### 4.2. Comparison with Other Denoising Methods

**Figure 8:**
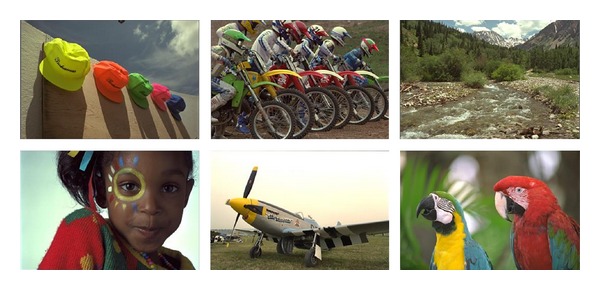
Some examples of the test images in Kodak losses true color image suite [20].

In order to further evaluate the proposed method, the proposed method is compared with other denoising methods. The test dataset is Kodak losses true color image suite which has 24 images. Some samples are shown in Figure 8.

The original images are corrupted by additive Gaussian white noise (AGWN). First, the RGB color image is mosaicked as bayer image, and then the AGWN is added. The standard deviation is *σ*_*n*_. In order to simulate the high SNR feature of green channel, the standard deviation of green channel *σ*_*ng*_ is lower than the other channel. In our experiment we set *σ*_*ng*_ = *σ*_*n*_ · *λ*, where *λ* is a constant between 0 and 1. Here we set *λ* = 0.8. The test is under two noise levels *σ*_*n*_ = 30 and *σ*_*n*_ = 60 which simulate the low noise and high noise.

CPSNR is employed as the objective criteria. CPSNR is defined as

(8) $$\begin{array}{c} \text{CPSNR}=10\log_{10}\left(\frac{L^{2}}{\text{MSE}}\right), \end{array}$$

where *L* is the dynamic range of the image. Here *L* = 255 for 8-bit pixel depth. MSE is the mean squared error between the original and distorted images **u**_1_ and **u**_2_:

(9) $$\begin{array}{c} \text{MSE}=\frac{\sum_{X=R,G,B}\sum_{K}{\left(u_{1}^{X}\left[k\right]-u_{2}^{X}\left[k\right]\right)}^{2}}{3N\cdot M}, \end{array}$$

where **k** is the spatial coordinates and the image size is *N* · *M*. A 20-pixel wide band around the border of the images is ignored when computing the CPSNR, since some of the tested algorithms do not perform well in side band. The pixel values are clipped to integers in [0–255] before computing the CPSNR.

For the compared methods, we ignore the complex algorithms, such as BM3D and K-SVD, since they cannot meet the requirements of digital imaging devices. Some simple and fast explicit filter kernel methods are selected as follows.

1. Wiener filter: it is a classical denoising method which is provided in Matlab imaging toolbox. The denoising of Wiener filter is executed on RGB color image, for it cannot be used directly on bayer pattern. The input noise image is demosaicked from the noise bayer image.
2. Joint demosaicking and denoising with space-varying filters (JDD) [16]: it is a typical demosaicking and denoising combined method which can be downloaded at author's website: http://www.danielemenon.netsons.org/pub/jdd/jdd.php. It works directly on bayer images.
3. Joint bilateral filter (JBF): it is similar to guided filter with the capability of merging extra information. So in the experiment, it uses green channel as guiding signal too. This method is implemented by us according to [21].

**Figure 9:**
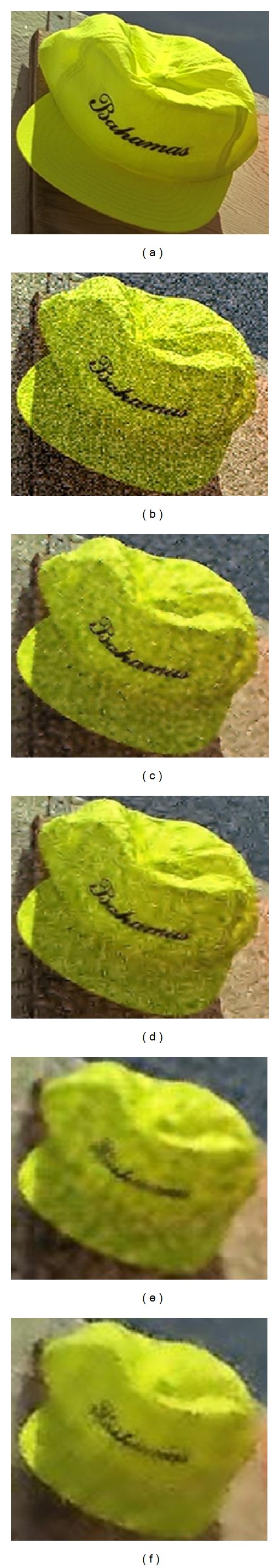
Denoising results of number 3 image in Kodak dataset with noise *σ*_*n*_ = 30: (a) original image; (b) noise image; and (c)–(f) images denoised by Wiener, JDD, JBF, and the proposed method, respectively.

For the parameters setting, the filter kernel size for all methods is 5 × 5. Wiener filter and JDD do not have any other parameters to set. Our method needs to set parameter *ε*, and JBF needs to set the geometric spread parameter *σ*_*d*_ and the photometric spread parameter *σ*_*r*_. Each parameter keeps the same for all the test images. We traverse all possible values for each parameter and take the best averaged CPSNR result for comparison. The test example is shown in Figure 9.

According to the visual inspection of Figure 9, our algorithm is superior to all others. The accurate CPSNR evaluation is shown in Tables 2 and 3.

From Tables 2 and 3, it can be concluded that the proposed method is significantly better than the RGB image based Wiener filter and the bayer image based filters JDD and JBF. The larger the noise is, the better result the proposed method has. The Wiener filter does the worst job, since it works directly on demosaicked RGB image where the noise characteristic is complicated. JBF is better than JDD because in the experiment JBF also joins green channel information to calculate the filter coefficients. Our algorithm is superior to JBF thanks to the usage of the advanced filter kernel [10]: guided filter.

## 5. Conclusions

This paper proposes a bayer image denoising method based on guided filter. The excellent properties of green channel in bayer pattern have been exploited. Green channel has the advantage of high sampling rate, which means more direct information of real world and less interpolation error. More importantly, it has the characteristic of higher ISO than others, which means higher SNR [18]. On the other hand, guided filter is an outstanding explicit filter kernel, which is better than bilateral filter. In our method, green channel is used as the guiding signal in guided filter. At first, the green values at red and blue locations are interpolated. Then the full-resolution green image is used to guide denoising by guided filter. It is worth noting that the green channel takes itself as guiding signal. The experiment on the actual bayer image demonstrates that green channel acts well as the guiding signal compared to other color channels. While compared with other explicit filter kernels, such as Wiener filter, JDD, and JBF, the proposed method is competitive. As for the methods that are based on block matching, domain transformation, and even the statistical learning or dictionary training, they suffer heavy computational burden and are not suitable for digital imaging devices. The further research aims at designing better green channel interpolation algorithm like demosaicking or searching for better guiding signal from bayer image than green channel.

## Figures and Tables

**Table 1:** Mean value of different color channels for Figure 3.

	*F*	L84	D50T	D65T	Natural
R	1.11	0.89	0.83	0.75	0.76
G	1.0	1.0	1.0	1.0	1.0
B	0.47	0.51	0.65	0.75	0.63

Green channel is regularized to 1.0.

**Table 2:** CPSNR (DB) comparison result with noise *σ*_*n*_ = 30.

Number	Input	Wiener	JDD	JBF	Proposed
1	18.86	21.25	23.05	23.32	**23.85**
2	19.58	22.55	25.68	26.38	**27.51**
3	19.28	22.29	25.26	26.31	**27.27**
4	19.15	22.19	24.95	25.85	**26.99**
5	19.16	21.36	22.28	**22.50**	22.43
6	19.06	21.60	23.90	24.32	**25.10**
7	19.11	21.95	24.01	24.00	**24.47**
8	18.78	20.57	22.24	21.98	**22.06**
9	18.94	21.92	24.55	25.06	**26.09**
10	18.96	21.96	24.69	25.41	**26.47**
11	19.23	21.92	24.10	24.53	**25.03**
12	19.07	22.16	25.18	26.15	**27.66**
13	18.93	21.17	22.56	22.83	**23.29**
14	19.06	21.69	23.07	**23.54**	23.42
15	19.81	22.86	25.73	26.36	**27.22**
16	19.04	21.91	24.94	25.94	**27.22**
17	19.40	22.25	24.72	25.43	**26.00**
18	19.35	22.13	24.09	24.48	**24.94**
19	18.97	21.56	24.09	24.12	**24.91**
20	20.33	22.83	25.20	25.33	**25.59**
21	18.96	21.66	23.72	24.11	**24.75**
22	19.01	21.84	24.28	24.86	**25.54**
23	19.25	22.27	25.12	25.74	**26.30**
24	18.96	21.71	23.63	24.10	**24.57**

Average	19.18	21.90	24.21	24.69	**25.36**

**Table 3:** CPSNR (DB) comparison result with noise *σ*_*n*_ = 60.

Number	Input	wiener	JDD	JBF	Proposed
1	13.61	16.48	19.55	19.94	**21.33**
2	14.46	17.35	21.70	21.86	**24.28**
3	13.94	16.93	21.03	21.55	**24.07**
4	13.93	16.96	21.01	21.49	**24.04**
5	14.16	16.73	18.92	19.24	**19.58**
6	13.82	16.63	20.21	20.64	**22.51**
7	13.73	16.70	20.02	20.28	**21.71**
8	13.82	16.33	18.54	18.50	**18.88**
9	13.54	16.61	20.27	20.72	**22.80**
10	13.50	16.57	20.36	20.98	**23.55**
11	13.93	16.76	20.16	20.61	**21.93**
12	13.80	16.85	20.98	21.47	**24.18**
13	13.83	16.53	19.27	19.68	**20.69**
14	14.00	16.80	19.85	20.27	**21.12**
15	14.77	17.47	21.52	21.71	**23.04**
16	13.69	16.69	20.83	21.41	**24.55**
17	14.11	16.91	20.68	21.23	**22.80**
18	14.33	17.11	20.61	20.86	**22.11**
19	13.70	16.54	20.05	20.29	**21.92**
20	15.02	17.24	20.59	20.64	**21.16**
21	13.62	16.55	19.85	20.32	**21.75**
22	13.76	16.75	20.49	20.95	**23.12**
23	14.04	16.98	20.95	21.29	**23.14**
24	13.78	16.69	20.01	20.52	**21.97**

Average	13.95	16.80	20.31	20.69	**22.34**
